# Design Consideration Investigation of Soft-Valve Pipe

**DOI:** 10.3390/mi13040568

**Published:** 2022-04-01

**Authors:** Xu Yang, Yiniu Luo, Chen Ji, Yugang Ren, Shizhen Li

**Affiliations:** Institute of Marine Science and Technology, Shandong University, Qingdao 266237, China; xu_y@sdu.edu.cn (X.Y.); luoyiniusues@163.com (Y.L.); jich@sdu.edu.cn (C.J.); ryg@ndsc.org.cn (Y.R.)

**Keywords:** silicone pipe, soft valve, configurations, parameters

## Abstract

This paper focuses on investigating the configuration and parameter selection of the silicone pipe in soft valve. According to the working principles of soft valve, five configurations and four structural parameters of silicone pipes are proposed and analyzed. The relationship between the pipe configuration and breakthrough pressure is investigated through experimental tests. The influence of the structural parameters on the breakthrough pressure is revealed by experiments as well. Based on the revealed design considerations, a three-way soft valve is designed, fabricated and tested. The experimental results show that the designed pipes have great stability and good sealability, which ensures the three-way soft valve possesses high breakthrough pressure. Finally, two application tests of the three-way soft valve are carried out, which further confirm the effectiveness of designed pipe and designed soft valve.

## 1. Introduction

Similar to snakes [1], fish [2] and many other creatures, soft robots can realize various bionic movements by their deformation, which enables them to grab complex objects, such as eggs [3,4], light bulbs [5], mushrooms [6] and coral reef [7], and perform many other flexible operations, such as human–machine interaction [8,9,10]. With specialized materials, a soft robot can also accommodate lots of external environments with characteristics such as high pressure [11], high temperature [12] or narrow space [13,14,15]. By virtue of the above advantages, soft robots have received a lot of attention in recent years. To realize high flexibility, good self-sensing capability, large driving capability and a compact structure, many soft robots are driven by hydraulic power [16,17,18]. For hydraulic transmission, the valve plays a crucial role in regulating the pressure and flow [19]. Nevertheless, the present hydraulic actuation is usually designed with hard valves, which decrease the flexibility of the soft robot. Hence, it is necessary to develop a soft control valve.

In the literature, many research efforts have been devoted to the development of soft control valves. For instance, Miyaki et al. designed a soft valve composed of flexible pipe and magnet that could control the movement of a pneumatic soft robot by self-excited vibration [20]. Luo et al. developed a soft kink valve, the opening and closing of which were controlled by the axial deformation of the silicone tube [21]. Rothemund et al. developed a soft bistable valve [22,23,24]. The interaction between the soft membrane and the silicone pipe can realize the opening and closing functions of the valve. As mentioned above, adjusting a soft valve relies on the deforming and folding motion of the silicone pipe. For investigating the working principle of a silicone pipe, Kamimura et al. proposed a numerical method for the two-dimensional deformation of a collapsible pipe [25]. Gent analyzed the elastic deformation and unstable state of a rubber pipe with theoretical modeling and experimental observation [26]. However, the on-and-off characteristics of the silicone pipe driven by elastomer are still unclear.

In this paper, the deforming and folding performance of the silicone pipes with various configurations and parameters are investigated in detail, which can provide several design guidelines for soft valves. Based on the revealed design considerations, a three-way soft valve is designed, fabricated, tested and applied. The rest of this paper is organized as follows. The Section 2 analyzes the main design factors of the soft-valve pipe. The Section 3 investigates the deformation process of the silicone pipe with various configurations and the influence of the pipe’s configuration on the breakthrough pressure. The deformation process of the silicone pipe with different structural parameters and the relationship between pipe structural parameters and breakthrough pressure are studied in the Section 4. On the basis of the above research results, a three-way soft valve is designed, fabricated and tested in the Section 5. The developed three-way soft valve is further applied in the motion control of two soft robots in the Section 5. Finally, a conclusion is made in the Section 6.

## 2. Design Consideration of Soft-Valve Pipe

The silicone pipe plays a significant role in a soft valve. To realize the opening and closing control of the soft-valve channel, the silicone pipe should be deformed in various shapes, which can result in different orifice areas. According to the throttling principle of the silicone pipe, the design consideration of soft-valve pipe is classified into two categories: pipe configurations and structural parameters.

**Figure 1:**
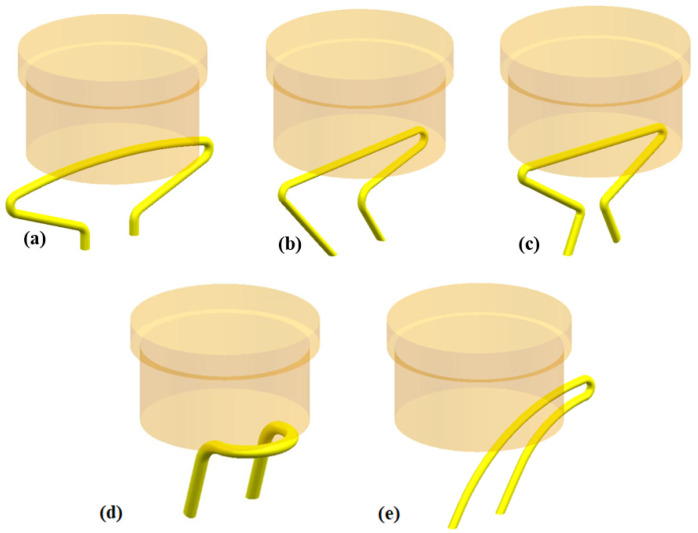
Five configurations of silicone pipe: (**a**) shape 1; (**b**) shape 2; (**c**) shape 3; (**d**) shape 4; (**e**) shape 5.

For silicone pipes, various configurations lead to different bending points, which further affect the throttle area of the soft valve. According to the deformation shapes of silicone pipes, the pipe configurations are classified into five types in this paper: shape 1, shape 2, shape 3, shape 4 and shape 5, as shown in Figure 1.

**Figure 2:**
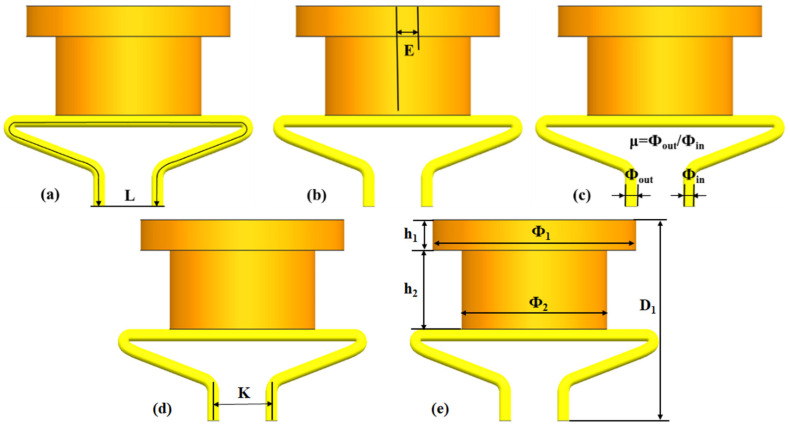
Four structural parameters of silicone pipe and several experimental parameters: (**a**) pipe length (L); (**b**) pipe eccentricity (E); (**c**) ratio of outer diameter to inner diameter (μ = Φ_out/_Φ_in_); (**d**) input–output ports space (K); (**e**) several experimental parameters.

For silicone pipes, various structural parameters result in different bending angles, which affect the throttle area of the soft valve as well. In this paper, the determination of four structural parameters is studied: pipe length (L), pipe eccentricity (E), the ratio of the outer diameter to the inner diameter (μ = Φ_out_/Φ_in_), and input–output ports space (K), as shown in Figure 2.

## 3. Pipe Configuration Investigation of Soft Valve

**Figure 3:**
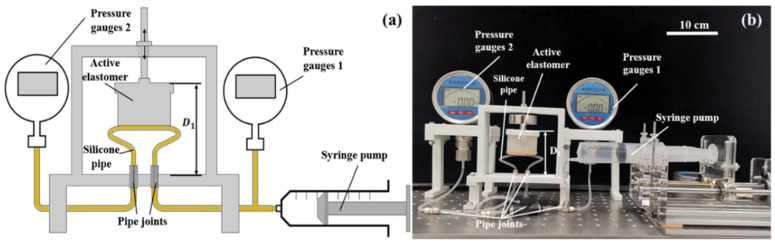
Experimental setup for pipe evaluation: (**a**) schematic; (**b**) photograph.

In order to investigate the effect of the pipe’s configuration, an experimental platform of the silicone pipe is set up, as shown in Figure 3. The experimental system consists of an active elastomer with position control capability, a syringe pump driven by a motor, two pressure gauges (0–1 MPa) and a silicone pipe (purchased from the Taizhou ChunShi New Material Co. Ltd. in Taizhou, China) to be tested. The active elastomer is fabricated from E660 silicone (purchased from the Shenzhen Hong Ye Jie Technology Co., Ltd. in Shenzhen, China) by pouring the silicone into a 3D-printed mold. The geometry parameters of the active elastomer are designed as: Φ_1_ = 56 mm, Φ_2_ = 50 mm, h_1_ = 9 mm and h_2_ = 31 mm. The breakthrough pressure of the silicone pipe can be identified in the following procedures: (1) initializing the input and output pressures of the silicone pipe to be zero; (2) increasing the input pressure of the silicone pipe by actuating the syringe pump; (3) identifying the peak value of the input pressure while the output pressure suddenly changes to larger than zero; (4) the peak value of the input pressure is assumed to be the breakthrough pressure of the silicone pipe.

**Figure 4:**
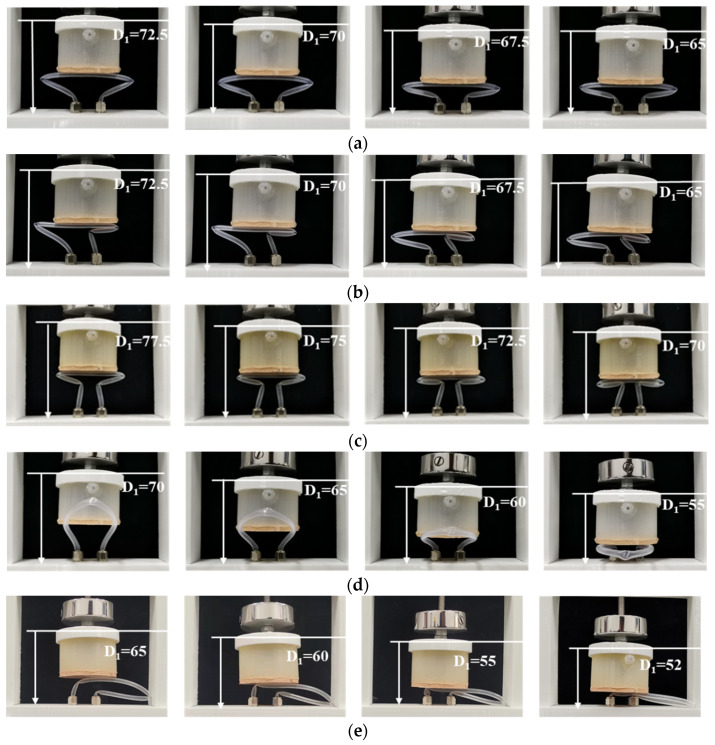
Experimental tests of five silicone pipes with different configurations: (**a**) deformation process of the shape 1 pipe; (**b**) deformation process of the shape 2 pipe; (**c**) deformation process of the shape 3 pipe; (**d**) deformation process of the shape 4 pipe; (**e**) deformation process of the shape 5 pipe.

By setting the pre-bending points, five different silicone pipes corresponding to the configurations shown in Figure 1 are fabricated. The breakthrough pressures of the five silicone pipes are evaluated under different deformations (different compression height D_1_). The regulation of compression height is implemented by controlling the position of the active elastomer (measured by a vernier caliper). The other parameters of the five silicone pipes are selected as L = 155 mm, E = 0 mm, Φ_out_ = 4 mm, Φ_in_ = 3 mm and K = 20 mm. The experimental test processes of the five silicone pipes are recorded and depicted in Figure 4. As can be seen from the photos, the fold of the silicone pipe becomes serve while decreasing the compression height D_1_, which is helpful for improving the breakthrough pressures.

**Figure 5:**
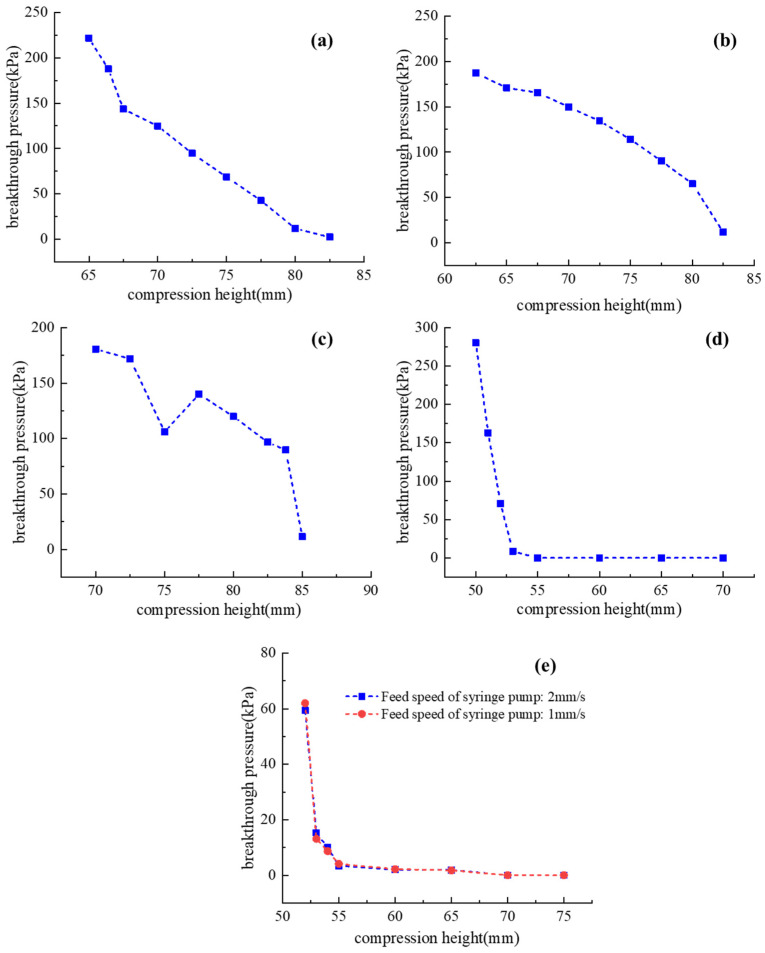
Breakthrough pressures of the five silicone pipes with different configurations: (**a**) shape 1 (set feed speed of pump: 2 mm/s); (**b**) shape 2 (set feed speed of pump: 2 mm/s); (**c**) shape 3 (set feed speed of pump: 2 mm/s); (**d**) shape 4 (set feed speed of pump: 2 mm/s); (**e**) shape 5 (set feed speed of pump: 1 mm/s and 2 mm/s).

Figure 5 depicts the measured breakthrough pressures of the five silicone pipes, as well as their variations along with the compression height D_1_. The feed speed of the syringe pump is set at 2 mm/s while identifying the breakthrough pressure. The experimental results show that the breakthrough pressures of silicone pipes decrease as the compression height D_1_ increases (for all five silicone pipes). For shape 3, shape 4 and shape 5, the decreasing curves are not stable. In contrast, the relationships between breakthrough pressure and compression height of shape 1 and shape 2 exhibit better linearity. Compared to shape 2, the pre-bending points of shape 1 are fewer, which can result in better stability and a simpler structure. Therefore, of the five pipe configurations, shape 1 is the best choice for the pipe design of the soft valve.

While the feed speed of the syringe pump is set at 2 mm/s, the rising time of the input pressure is observed to be 12.3 s (shape 5 pipe, from 0 kPa to 50 kPa). To further investigate the influence of the pressure-ramping rate on the breakthrough pressure, the breakthrough pressure of the shape 5 pipe is further measured when the feed speed of the syringe pump is set at 1 mm/s, as shown in Figure 5e. The rising time of input pressure is observed to be 25.8 s (shape 5 pipe, from 0 kPa to 50 kPa). As can be seen from the figure, a slight difference exists between the two breakthrough pressure curves. Therefore, the influence of pressure ramping rate on the breakthrough pressure is not obvious.

## 4. Pipe Parameter Investigation of Soft Valve

**Figure 6:**
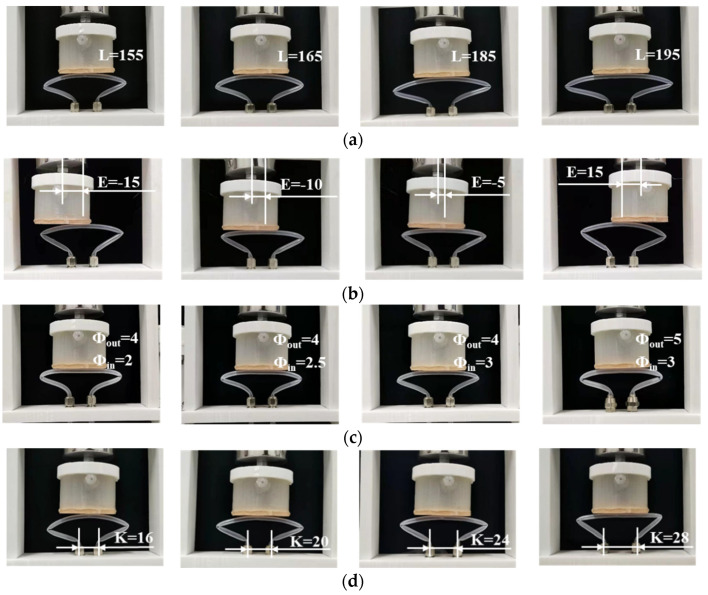
Experimental tests of the silicone pipe with different structural parameters: (**a**) different lengths L (E = 0 mm, Φ_out_ = 4 mm, Φ_in_ = 3 mm, K = 20 mm, D_1_ = 75 mm); (**b**) different eccentricity E (L = 155 mm, Φ_out_ = 4 mm, Φ_in_ = 3 mm, K = 20 mm, D_1_ = 75 mm); (**c**) different ratios of outer to inner diameters μ (L = 155 mm, E = 0 mm, K = 20 mm, D_1_ = 75 mm); (**d**) different input–output ports spaces K (L = 155 mm, E = 0 mm, Φ_out_ = 4 mm, Φ_in_ = 3 mm, D_1_ = 75 mm).

Based on the experimental platform shown in Figure 3, the effect of the pipe’s structural parameters is further investigated. For better performance and contrastive analysis, the silicone pipes are designed using shape 1 in this section. Figure 6a depicts the experimental test process of the silicone pipes with different pipe lengths L. Figure 6b depicts the experimental test process of the silicone pipes with different pipe eccentricity E. Figure 6c depicts the experimental test process of the silicone pipes with different ratios of outer to inner diameters μ (μ = Φ_out_/Φ_in_). Figure 6d depicts the experimental test process of the silicone pipes with different input–output ports space K. As can be seen from the figures, the four structural parameters all contribute to the bending angle of the silicone pipe.

**Figure 7:**
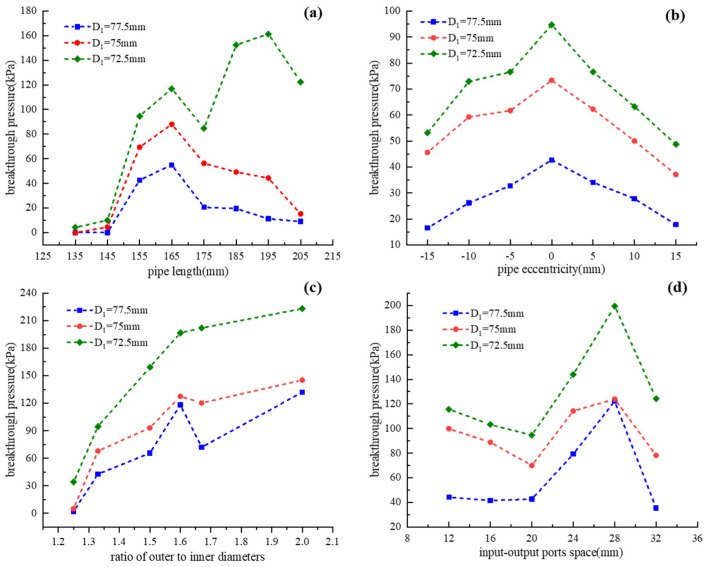
Breakthrough pressures of the silicone pipes with different structural parameters: (**a**) pipe length; (**b**) pipe eccentricity; (**c**) ratio of outer to inner diameters; (**d**) input–output ports space.

The breakthrough pressures of the silicone pipes with different structural parameters are recorded and depicted in Figure 7. It can be seen from Figure 7a that the breakthrough pressure increases firstly and then decreases with the increase in the pipe’s length. With the increase in pipe eccentricity, the breakthrough pressure first increases and then decreases, as shown in Figure 7b. As can be seen from Figure 7c, the breakthrough pressure gradually increases with the increase in the ratio of outer to inner diameters. As shown in Figure 7d, the breakthrough pressure fluctuates with the increase in input–output ports spaces. Therefore, the breakthrough pressure of the silicone pipe can be designed by selecting appropriate values of pipe length (L), pipe eccentricity (E), the ratio of outer to inner diameters (μ) and input–output ports space (K).

## 5. Development and Application of the Three-Way Soft Valve

### 5.1. Development of the Three-Way Soft Valve

**Figure 8:**
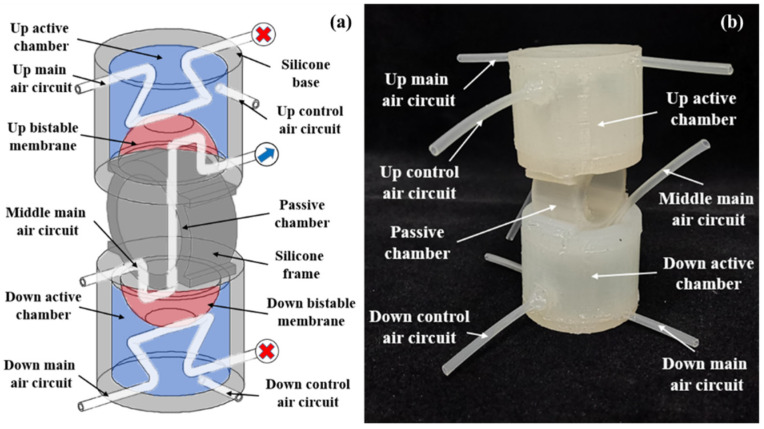
Three-way soft valve: (**a**) schematic; (**b**) prototype.

Based on the throttling principle and performance of the silicone pipe, a three-way soft valve [27] is designed in this section, as shown in Figure 8a. The valve consists of an up active chamber, a down active chamber and a passive chamber. The two active chambers have the same structures: bistable membrane, control air circuit, main air circuit and silicone base. The passive chamber is designed with a middle main air circuit and silicone frame. A three-way soft valve prototype is fabricated, as shown in Figure 8b. The bistable membrane and air circuits are both made of silicone material.

**Figure 9:**
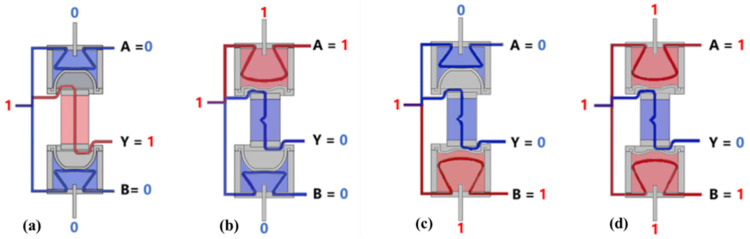
Four working states of the three-way soft valve: (**a**) state 1; (**b**) state 2; (**c**) state 3; (**d**) state 4.

The three-way soft valve is designed with four logic working states, as shown in Figure 9. State 1: If the up and down control air circuits are not pressurized, the up and down main air circuits will be closed, and the middle main air circuit will be opened. State 2: If the up control air circuit is pressurized and simultaneously the down control air circuit is not pressurized, the up main air circuit will be opened, the middle and down main air circuits will be closed. State 3: If the up control air circuit is not pressurized and simultaneously the down control air circuit is pressurized, the down main air circuit will be opened, the up and middle main air circuits will be closed. State 4: If the up and down control air circuits are both pressurized, the up and down main air circuits will be opened, the middle main air circuit will be closed.

**Figure 10:**
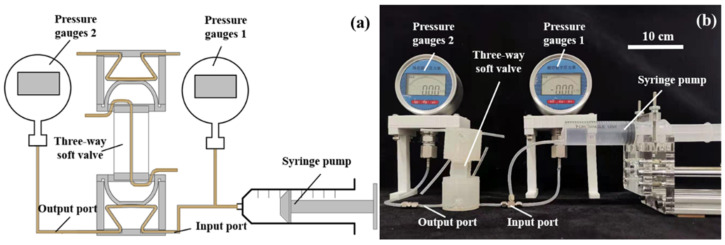
Experimental setup for the three-way soft valve: (**a**) schematic; (**b**) photograph.

For evaluating the performance of the three-way soft valve, another experimental platform is set up, as shown in Figure 10. The experimental system consists of a syringe pump driven by a motor, two pressure gauges and the three-way soft valve to be tested. The syringe pump is employed to provide a stable air source. The two pressure gauges are used to measure the input and output pressures of the main air circuits of the three-way soft valve.

**Figure 11:**
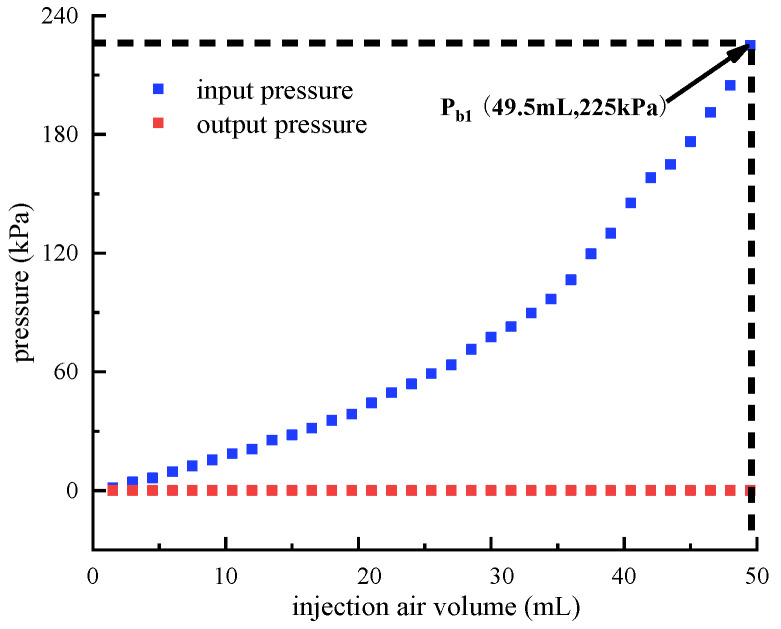
Breakthrough pressure identification of the up main air circuit. (the up and down control air circuits are not pressurized.).

While the up and down control air circuits are not pressurized, the breakthrough pressure of the up main air circuit is identified. Figure 11 depicts the measured input and output pressures of the up main air circuit. It is observed that the input pressure increases from 0 kPa to 225 kPa as the volume of injection air improves from 0 mL to 49.5 mL. Even the input pressure reaches 225 kPa, the output pressure remains at 0 kPa. Therefore, the breakthrough pressure of the up main air circuit is larger than 225 kPa.

**Figure 12:**
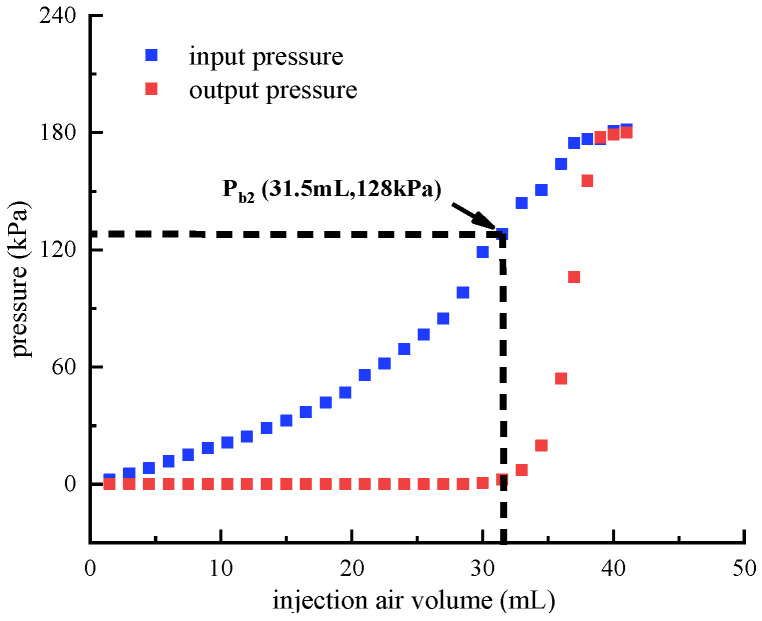
Breakthrough pressure identification of the middle main air circuit. (The up control air circuit is pressurized, and the down control air circuit is not pressurized.).

While the up control air circuit is pressurized and the down control air circuit is not pressurized, the breakthrough pressure of the middle main air circuit is identified. Before the test, the up control air circuit is pressurized by the syringe pump and then blocked, which ensures that the up bistable membrane is reversed. Afterwards, the syringe pump is used to pressurize the input port of the middle main air circuit. Figure 12 depicts the resulting input and output pressures of the middle main air circuit. As can be seen from the figure, while the input pressure increases to 128 kPa, the output pressure suddenly increases. Therefore, the breakthrough pressure is obtained to be 128 kPa.

**Figure 13:**
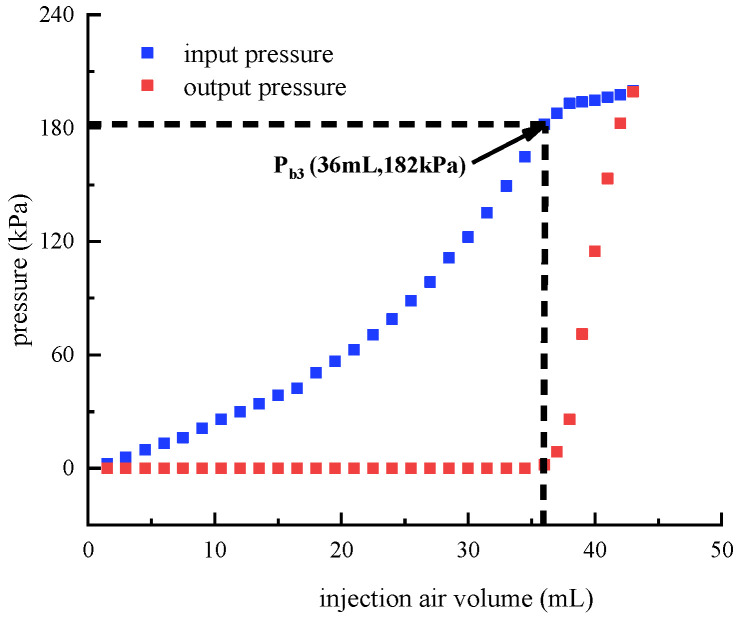
Breakthrough pressure identification of the middle main air circuit. (The up and down control air circuits are both pressurized.).

When both the up and down control air circuits are pressurized, the breakthrough pressure of the middle main air circuit is further identified. Before the test, the up and down control air circuits are pressurized by the syringe pump and then blocked, which ensures that the up and down bistable membranes are both reversed. Afterwards, the syringe pump is used to pressurize the input port of the middle main air circuit. Figure 13 depicts the resulting input and output pressures of the middle main air circuit. It can be observed that the output pressure begins to increase when the input pressure reaches 182 kPa. Therefore, the breakthrough pressure is 182 kPa. The additional reversed bistable membrane helps to improve the breakthrough pressure of the middle main air circuit.

### 5.2. Application of the Three-Way Soft Valve

**Figure 14:**
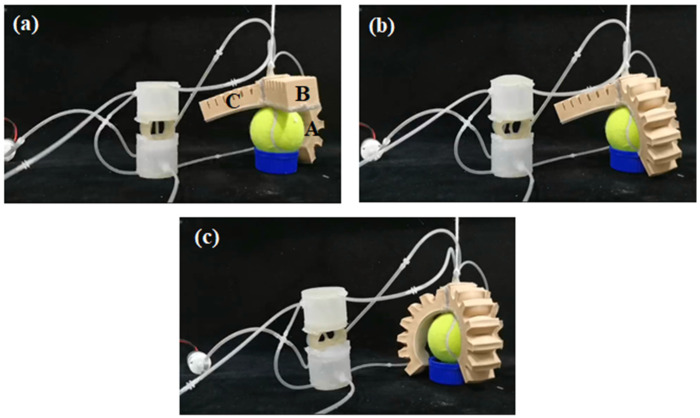
State of the pneu-net gripper while the three-way soft valve is in: (**a**) state 1; (**b**) state 2; (**c**) state 3.

To evaluate the practical applicability of the three-way soft valve, the developed soft valve prototype is used to control two types of soft robots: pneu-net gripper [18] and corrugated crawling robot [28]. First, the three-way soft valve is applied to a soft gripper with three pneu-net fingers: the input ports of the three main air circuits are connected to a micro-air pump (type: SC3710PM, maximum flow: 2.8 L/min, maximum pressure: 80 kPa), the output port of the up main air circuit is connected to the B finger, the output port of the middle main air circuit is connected to the A finger and the output port of the down main air circuit is connected to the C finger. Figure 14a shows the grasping operation of the pneu-net gripper, while the three-way soft valve is in state 1. As the middle main air circuit is opened, the output air of the pump will be provided to the A finger, resulting in the bending state of the A finger. Then, the three-way soft valve is switched to state 2, as shown in Figure 14b. It is observed that the A finger is still pressurized even when the middle main air circuit is closed. In addition, high-pressure air will be provided to the B finger through the up main air circuit, causing the B finger to bend. Afterwards, the three-way soft valve is switched to state 3, as shown in Figure 14c. The C finger is pressurized by the high-pressure air from the down main air circuit. In the meantime, A finger and B finger are still pressurized. Finally, the three pneu-net fingers are all in bending shape, which successfully accomplishes the grasping operation.

**Figure 15:**
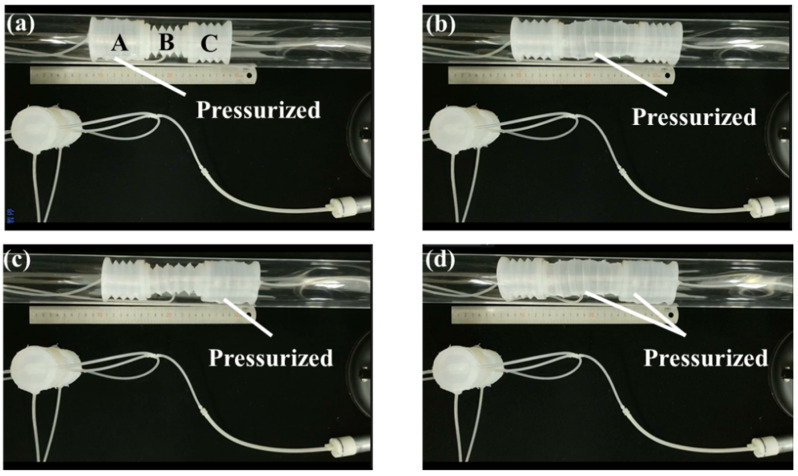
State of the corrugated crawling robot while the three-way soft valve is in: (**a**) state 1; (**b**) state 2; (**c**) state 3; (**d**) state 4.

The three-way soft valve is then applied to a corrugated crawling robot: the input ports of the three main air circuits are connected to a micro-air pump (type: SC3710PM, maximum flow: 2.8 L/min, maximum pressure: 80 kPa), the output port of the up main air circuit is connected to the B chamber of the crawling robot, the output port of the middle main air circuit is connected to the A chamber of the crawling robot and the output port of the down main air circuit is connected to the C chamber of the crawling robot. Different from the pneu-net gripper, the three chambers of the corrugated crawling robot are designed with micro-holes that can release air. The chamber will only be pressurized when the air source is being provided (the corresponding main air circuit is opened). Figure 15a shows the motion state of the crawling robot while the three-way soft valve is in state 1. The A chamber of the crawling robot is pressurized by the air from the middle main air circuit. After that, the three-way soft valve is switched to state 2, resulting in the crawling state shown in Figure 15b. The B chamber of the crawling robot is pressurized by the air from the up main air circuit. Afterwards, the three-way soft valve is switched to state 3, resulting in the crawling state shown in Figure 15c. The C chamber of the crawling robot is pressurized by the air from the down main air circuit. Finally, the three-way soft valve is switched to state 4, resulting in the crawling state shown in Figure 15d. The B and C chambers of the crawling robot are pressurized by the air from the up main air circuit and the down main air circuit, respectively. With the above crawling states, the corrugated crawling robot successfully accomplishes the crawling motion.

## 6. Conclusions

This paper studies the deformation characteristics and design considerations of a soft-valve pipe. For the soft-valve pipe, several design considerations were analyzed and summarized. The effects of the pipe’s configurations and the pipe’s structural parameters on the pipe deformation and breakthrough pressure were experimentally studied. The application experiments of the silicone pipes were carried out as well. Based on the above results, the following three conclusions are drawn.

(1) Of the five pipe configurations, shape 1 and shape 2 have a better linear relationship between breakthrough pressure and compression height. Compared to shape 2, shape 1 also exhibits a simpler and more stable structure.
(2) The breakthrough pressure of the silicone pipe in the soft valve can be designed by selecting the appropriate values of the pipe length, pipe eccentricity, ratio of outer diameter to inner diameter and input–output ports space.
(3) By virtue of reasonable pipe configuration and pipe parameter, the developed three-way soft valve shows good air circuit controllability and can be applied to control the states of a pneu-net gripper and a corrugated crawling robot.

## 7. Patents

Patent authorized: Y. Luo; C. Ji; S. Li, et.al. Small pneumatic three-way soft valve. Authorized 4 January 2022, CN113007384B.

## Data Availability

Not applicable.
